# What Is the Mechanism of Government Green Development Behavior Considering Multi-Agent Interaction? A Meta-Analysis

**DOI:** 10.3390/ijerph19148263

**Published:** 2022-07-06

**Authors:** Xingwei Li, Jiachi Dai, Xiaowen Zhu, Jinrong He, Jingru Li, Xiang Liu, Yicheng Huang, Qiong Shen

**Affiliations:** 1 College of Architecture and Urban-Rural Planning, Sichuan Agricultural University, Chengdu 611830, China; xwl@sicau.edu.cn (X.L.); 2020325016@stu.sicau.edu.cn (J.D.); hejinrong@stu.sicau.edu.cn (J.H.); 2021325022@stu.sicau.edu.cn (J.L.); liuxiang@stu.sicau.edu.cn (X.L.); huangyicheng@stu.sicau.edu.cn (Y.H.); 2 School of Management, Jiangsu University, Zhenjiang 212013, China; xiaowen.zhu@cranfield.ac.uk; 3 Centre for Design, Cranfield University, Cranfield, Bedford MK43 0AL, UK

**Keywords:** government, green development, environmental regulation, government supervision, multi-agent, meta-analysis

## Abstract

Worsening environmental problems have created more and more challenges for green development, and the government is often seen as an important guide in turning this situation around. A government generally enacts green development through green development behavior, but previous research has not revealed the mechanism of this behavior. In addition, the multi-agent interaction between the government and green development behavior also needs to be explored. Based on an integrated theoretical model, the authors of this study adopted a meta-analysis method to analyze 18 high-quality published pieces from 6 mainstream databases and described the mechanism of government green development behavior in exploring and thinking about multi-agent interactions. In addition, the authors of this study explored differences in the roles of central and local government green development behaviors and the moderating role of regional heterogeneity. The research results showed that: (1) Enterprise economic behavior, enterprise environmental behavior, enterprise social behavior, and public participation are all significantly positively affected by government green development behavior; (2) local government green development actions have stronger effects than central government actions; (3) regional heterogeneity moderates the effect of government green development behavior. Furthermore, the authors of this study propose relevant countermeasures and suggestions from the government’s point of view. This research provides a theoretical and practical reference for governments to better improve their environmental systems and environmental supervision.

## 1. Introduction

**Figure 1:**
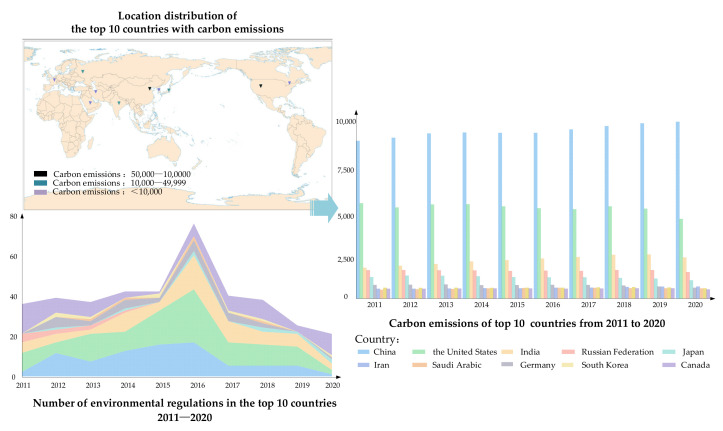
Carbon emissions and environmental regulations.

Driven by economic development and population growth [1], the carbon emission situation is not optimistic. Global carbon dioxide emissions rebounded in 2021, rising by 6.0% compared to 2020 [2]. In response, governments in many countries have actively enacted legislation to curb this situation. The number of environmental regulations and carbon emissions from 2011 to 2020 obtained by [3,4] are shown in Figure 1. In addition to the problem of increasing carbon emissions, society is also caught in a worrying environmental situation featuring water and solid waste pollution. It has been shown that 80% of global wastewater is discharged directly into the environment without treatment [5]. In terms of waste, the World Bank stated that global solid waste is expected to reach 3.40 billion tons in 2050 [6]. The unbalanced development of solid waste resource disposal and recycling rate has also made integrated solid waste management a heavy burden for society. However, against the background that various countries and regions are gradually paying attention to environmental issues, the complex and dynamic characteristics of social systems have generated more and more challenges for the government to face in managing environmental issues. According to the report released by the United Nations Environment Programme in 2021 [7], it is expected that the governments involved in the 2030 Paris Agreement will still not be able to achieve their target carbon emission reductions. Implementing green development behavior is a key move for governments to reverse this situation. A government can adopt incentive-oriented or mandatory-oriented green development behaviors to improve the green development level of its region. The subjects in a social system are interconnected and mutually reinforcing. When these subjects face government behavior, they also show different coping strategies, such as the green transformation of enterprises, the greenwashing of enterprises, increasing public green demand, and market greening. Therefore, how government green development behavior affects other social subjects is a topic worthy of discussion.

Based on relevant studies [8,9,10,11,12], the authors of this study defined governmental green development behavior as a behavior enacted by the government to achieve green development and economic growth. Researchers have previous conducted the following types of studies on the topic of governmental green development behavior. First is environmental regulation-related study. This type of research mainly focuses on the driving role of environmental regulation orientation (command-and-control, market-based, and voluntary) and the nature of rewards and punishments on the behavior of enterprises such as green technology innovation [13,14,15]. Second is environmental regulation study. This type of research explores the impact of government regulation on the environmental behavior of enterprises, the public, and other subjects [16]. Third is regulatory effect study. For example, based on panel data, Ji et al. [17] showed that both direct and indirect government support will enhance the direct linkage effect of environmental CSR and collaborative innovation. Overall, previous studies have achieved significant results and have important academic value. However, the following research gaps still exist. First, the driving path is not yet clear. Previous studies have mainly been conducted with non-governmental subjects such as enterprises [18], industries [19], and the public [15], and the interactions between them have not been explored from the governmental perspective. Additionally, there has been heterogeneity in the role of positive, negative, and no effects. Second, systematic studies on governmental green development behaviors are still lacking. Third, the differences in the role of green development behavior between local and central governments need to be explored. Accordingly, the authors of this study selected 18 empirical studies related to government green development behavior during 2012–2021 for a meta-analysis based on stakeholder and neo-institutional theory perspectives. In this way, the interactions between local government, central government, enterprises, and the public were explored, and the mechanism of the role of government green development behavior was revealed to answer the above-mentioned shortcomings.

Considering the realistic research background and current situation of theoretical research development, the core question of this study can be proposed: what is the mechanism for considering the role of multi-body governmental green development behavior? The innovation of this study is that it enriches the study of green development behavior from the government’s perspective and provides more theoretical explanations for the differences between central and local governments. In the context of government-led enterprise action and public participation in green development, this research provides theoretical and practical reference for governments to better improve environmental systems and environmental regulation. In addition, this study provides a new perspective for thinking about the development of the circular economy.

This study is structured as follows. Section 2 presents an integrated hypothesis model based on the theories and literature review. Section 3 presents a description of the meta-analysis process. Section 4 shows the results of the meta-analysis of main effects, subgroup and moderating effects, and discussions and management insights on the results. Section 5 summarizes the findings and presents the limitations of the study, as well as future research directions.

## 2. Theoretical Basis and Hypothesis Development

### 2.1. Theoretical Basis

Stakeholder theory assumes that individuals or groups that directly or indirectly influence or are influenced by organizational goals are stakeholders [20]. Stakeholder theory has clear requirements in terms of both the subject of interest and the object of concern: focus on the overall interest and emphasize both economic and social interests. Since Freeman et al. [20] first systematically elaborated stakeholder theory, it has been used to emphasize the focus on how to achieve the overall interests of stakeholders rather than individual interests in the process of business management. In addition, the theory encourages business managers to abandon the economic-first view and to consider the social benefits brought by the business instead. Nowadays, the theory is an multidisciplinary intersection point of environmental science [21], economics [22], and sociology [23]. Inspired by this theory, the relevant groups involved in the practice of governmental green development behavior can be analyzed and discussed in depth as stakeholders to understand the interactions between the relevant parties. Researchers have explored the stakeholders of government green development behavior from different perspectives. Du et al. [24] explored the decision-making behavior of three stakeholders—the government, public, and enterprises—under different scenarios using evolutionary games from the perspective of construction waste management. Mia et al. [25] studied the impact of changes in government regulation on enterprises’ resource disclosure of environmental information based on the use of stakeholder theory and legitimacy theory. In this regard, the authors of this study considered the government, public, and enterprise as the significant stakeholders of government green development behavior from a social system perspective.

Government is the expression of an institutionalized political model [26]. In other words, governmental green development behavior is inextricably linked to institutional environment. Essentially, at the heart of neo-institutional theory is the binding effect of social structures acting on organizations. This binding effect manifests itself in three different forms: coercive pressure, normative pressure, and imitative pressure [27]. It follows that, unlike institutional theory, neo-institutional theory places more emphasis on institutional diversity. As mentioned earlier, from the neo-institutional theory perspective, governmental green development behavior acts as coercive and normative institutional pressure on the public and enterprises. When the public and enterprises are constrained by these two types of pressures, they will respond by exercising their subjective initiative as much as possible, e.g., in the form of obedience, resistance, and decoupling [28]. Previous studies have also explored the response of governmental green development behavior to the drive of the public and enterprises. Li et al. [8] revealed the driving path of environmental regulation on the implementation of green technology innovation in construction enterprises by constructing a vector autoregressive model. Chen et al. [29] revealed the driving mechanism of environmental law on public environmental behavior based on the theory of planned behavior. It is not difficult to find that when the public and enterprises face institutional pressure from governmental green development behavior, they usually choose to adopt or resist the proposed strategy.

**Figure 2:**
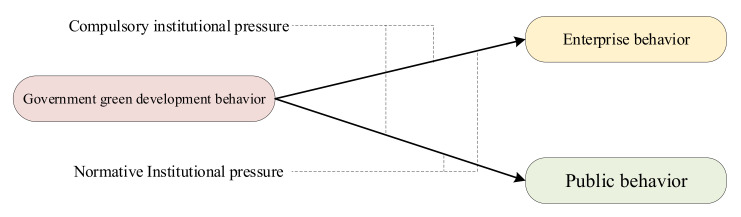
Integration theory model.

In summary, the authors of this study analyzed government green development behavior from the perspective of the specified subjects and their interactions. Based on stakeholder theory, the authors of this study considered the government, enterprises, and the public as the three stakeholders of government green development behavior. Then, the authors of this study analyzed the interactions between government green development behavior, enterprises, and the public based on neo-institutional theory. Thus, the authors of this study built an integrated theoretical model of governmental green development behavior around stakeholder theory and neo-institutional theory, as shown in Figure 2. The differences between this study and other similar studies are shown in Table 1.

### 2.2. Hypothesis Development

#### 2.2.1. Government Green Development Behavior and Enterprise Behavior

Triple bottom line theory suggests that enterprise performance can be measured in terms of economic, environmental, and social aspects [35]. Accordingly, the authors of this study divided enterprise behavior into enterprise economic behavior, enterprise environmental behavior, and enterprise social behavior. Enterprise economic behavior is mainly expressed as enterprise profitability, Agan et al. [31] used structural equation modeling to reveal the significant drive of environmental regulation on the expected returns of small and medium enterprises in Turkey. In terms of enterprise environmental behavior, previous studies mostly focused on enterprise green innovation behavior. Wu et al. [36] used least partial squares to analyze the significant positive driving effect of coercive environmental regulations on exploratory and developmental environmental innovation technologies of manufacturing enterprises in Taiwan, China. Scholars have mainly considered enterprise social behavior through the lens of corporate social responsibility. Zhang et al. [37] used a quasi-natural experiment to study listed enterprises in a pilot city in China, and they showed that environmental policies positively influence state-owned enterprises’ sense of social responsibility. The implementation of environmental regulations introduced by a central government has shown uneven effects on different local governments [38].

#### 2.2.2. Government Green Development Behavior and Public Behavior

The huge green demands of the public drive the process of green development as external political pressure [39]. As a group, the public is involved in green development in various forms, e.g., in the form of green consumption, public opinion monitoring, media regulation, and monitoring by non-governmental environmental agencies. Al-Swidi et al. [40] studied the factors influencing green consumption among Qatari youth using least squares structural equation modeling, and they showed that government green development behavior has a direct or indirect impact on green consumption. Xu et al. [41] analyzed the evolution from environmental authoritarianism and consultative authoritarianism to collaborative regulation between the Chinese government and environmental non-governmental organizations during the period of 1990–2020. Walther et al. [42] argued that the government and environmental non-governmental organizations are jointly involved in using media coverage and social network propaganda to raise public awareness of green issues.

#### 2.2.3. Local Government and Central Government Green Development Behavior

In the continuous and efficient promotion of green development, central and local governments play important guiding roles and increase environmental governance. In terms of local governments, Wu et al. [43] found that local government green development behaviors such as environmental legislation and regulation can contribute to improvements of regional environmental quality. In terms of central governments, Wang et al. [44] studied the environmental governance capacity of central environmental monitoring mechanisms based on a regression discontinuity design model, and the results of the study showed that central environmental monitoring mechanisms are effective in environmental governance. However, the two kinds of government often play different roles in the face of different environmental powers and responsibilities. Zhou et al. [45] found significant gaps between the two kinds of government regarding the application of green investment approaches in the implementation of climate policies through a binary instrument study.

#### 2.2.4. Moderating Effects of Regional Heterogeneity

Wang et al. [44] found variability in the regional green development levels corresponding to 30 provinces in China using the entropy weight method, indicating that different regions have different roles in the level of governmental green development. Additionally, Zou et al. [46] found that improving the level of regional green development would promote the green development of the surrounding regions.

**Figure 3:**
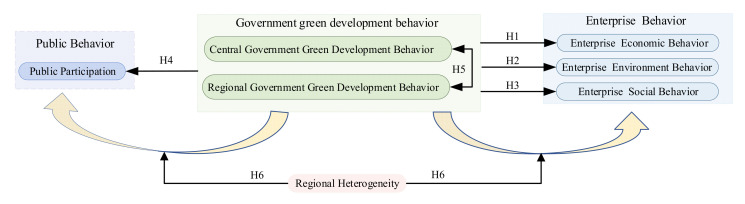
Hypothetical framework.

In summary, the authors of this study propose the following research hypotheses. The hypothetical framework is shown in Figure 3.

**H1.**  *Government green development behavior significantly and positively influences the economic behavior of enterprises.*

**H2.**  *Government green development behavior significantly and positively influences the environmental behavior of enterprises.*

**H3.**  *Government green development behavior significantly and positively influences the social behavior of enterprises.*

**H4.**  *Government green development behavior significantly and positively influences public participation.*

**H5.**  *The effect of local government green development behavior is stronger than that of central government green development behavior.*

**H6.**  *Regional heterogeneity can moderate the degree of the government green development behavior.*

## 3. Method and Data

### 3.1. Meta-Analysis

Meta-analysis is based on predetermined mathematical criteria for the statistical analysis of high-quality research [47], and it has been widely used in many fields such as medicine [48], environmental science [49], and organizational behavior [9]. The main reasons for the authors of this study to carry out research on the mechanism of the role of government green development behavior based on meta-analysis are as follows. Firstly, the mechanism of the role of government green development behavior is still unclear, and there have been some differences in the findings of existing empirical studies on the strength and significance of this behavior. Secondly, compared to traditional methods, meta-analysis strictly follows the screening criteria and quantitative analysis means, which makes the corresponding research results more objective. Therefore, the authors of this study focused on the green development behavior of governments as the object and collected empirical research on its relationship with enterprises and the public from an objective perspective. Furthermore, the authors of this study analyzed the mechanism of government green development behavior by using the quantitative means of meta-analysis, ultimately providing a reference for governments to improve green development.

#### 3.1.1. Data and Code

To ensure more representative and comprehensive data, the authors of this study searched the core collection of the Web of Science database, Elsevier Science Direct, Springer, Nature, Wiley, and Emerald. “Environmental policy”, “government environmental behavior”, “government environmental supervision”, and “government environmental subsidy” were selected as the subject terms, and the literature was searched from 2012 to 2021. The title/keyword/abstract of the literature had to include but was not limited to the above-mentioned subject terms. In summary, a total of 2312 documents were collected for this study. The search was conducted on 1 June 2022.

**Figure 4:**
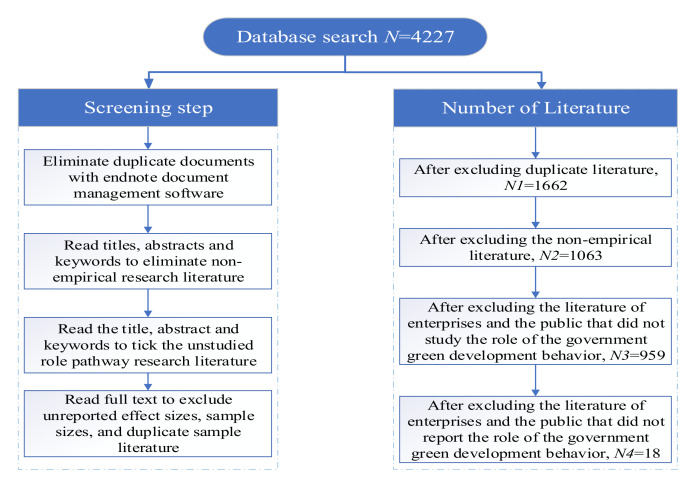
Sample literature screening process.

Furthermore, the authors of this study screened the literature with the following screening criteria to obtain studies that fit the objectives: (1) The target literature had to be empirical studies; (2) the target literature had to consider whether government green development behavior affects businesses/public; (3) the target literature had to report the sample size and effect size; and (4) the sample involved in the target literature had to be an independent sample. The specific screening steps are shown in Figure 4. Finally, a total of 18 studies were included in this study as the target sample literature.

Effect sizes are often used as research indicators for meta-analyses to measure the strength of effect values [50]. Considering that the effect size indicators chosen for different studies may differ, the effect size needs to be transformed during the research process. Accordingly, it was observed that most effect sizes output by the target literature were Pearson correlation coefficients. However, the Pearson correlation coefficient, as an isometric scale, cannot simply be averaged. Therefore, in this study, Fisher’s Z was used as the effect size indicator. Based on the findings of Fisher [51], the transformation formula is as follows:

(1) $$Z=0.5\times \ln(\frac{1+r}{1-r})$$

(2) $$\mathrm{SEz}=\sqrt{\frac{1}{n-3}}$$

Depending on the purpose of the study and the content of the literature, the authors of this study divided the coding content into two parts: basic and statistical information. The basic information included first author, year of publication, and study variables. The statistical information included the sample size and the transformed effect size (Fisher’s Z). Based on the coding results (as shown in Table 2), it can be seen that this study involved 18 effect sizes and 10,782 samples. Of the studied papers, the sample size of Liu et al. (2021) [34] was 6309, accounting for 58.51% of the total sample size.

#### 3.1.2. Publication Bias Test

**Figure 5:**
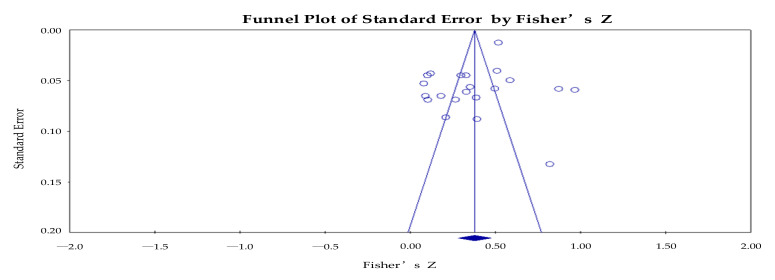
Total sample funnel chart.

Unpublished literature, non-English literature, and gray literature were not included in this study because their use could have led to a situation of publication bias in the findings. Therefore, the authors of this study utilized funnel plots, Rosenthal’s fail-safe N, Begg and Mazumdar rank correlation, and Egger’s regression intercept methods to test for bias. First, the funnel plot was output using Comprehensive Meta-Analysis 3.0 (CMA3.0) software to perform a preliminary test of the target literature. As seen in Figure 5, the target literature was not perfectly symmetrically distributed. This was a preliminary indication of possible bias in this study.

Next, the literature was further tested using CMA 3.0, and the results are shown in Table 3. Rosenthal’s fail-safe N test showed that the z-value was greater than 1.96, *p* was less than 0.001, and α = 0.05. It can be concluded that there was no publication bias in this study. The Begg and Mazumdar rank correlation test showed that the *p*-values of the two-sided tests were all greater than 0.05 and did not reach significance. This indicates that there was no publication bias in this study. Based on the results of Egger’s regression test, the *p*-values of the two-sided test were greater than 0.05 and did not reach significance. This shows that there was no publication bias in this study, and these overall results indicate that there was no bias in this study.

#### 3.1.3. Heterogeneity Test

Considering the differences in the design of research methods and sources of research data used in the target literature, it was extremely necessary to test the heterogeneity of the studied literature. In this study, Q value and I^2^ were used to test the heterogeneity of the studies [62], and the results of the test are shown in Table 4. As can be seen from the table, the Q test had a *p*-value < 0.001 and Q-value > degrees of freedom (df). This indicates significant heterogeneity in this study. The test results showed that the I^2^ value was 95.625%, indicating that 95.625% and 4.375% of the observed variance in this study were caused by the true number of variances and random errors in the effect sizes, respectively. According to [62], an I^2^ value in the 75–100% range would indicate a high degree of heterogeneity in this study, and a τ^2^ value of 0.047 would indicate a 3.7% variance between studies. In summary, the authors of this study adopted a random effects model for analysis to cope with the heterogeneity between studies.

#### 3.1.4. Outlier Test

**Figure 6:**
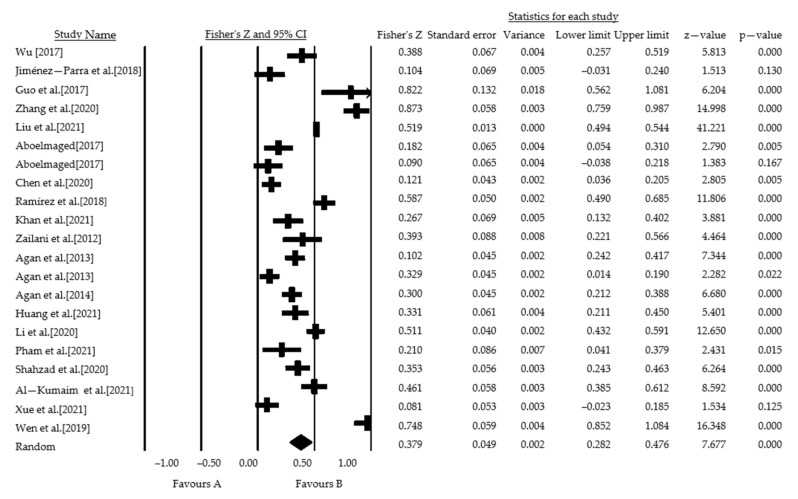
Total sample forest plot.

The forest plot output from the meta-analysis based on the random effects model showed (see Figure 6) that three studies clearly crossed the zero cut-off, identifying the work of Jiménez-Parra et al. (2018) [52] on enterprise environmental behavior, Aboelmaged (2017) [54] on enterprise social behavior, and Xue et al. (2021) [60] as outliers. Therefore, these studies were excluded from this study.

#### 3.1.5. Sensitivity Analysis

In this study, the robustness of the findings was tested by excluding one study at a time as a way to exclude anomalous studies. In the enterprise economic behavior dimension, the effect sizes did not significantly vary within a range of 0.120 (i.e., the effect sizes in this dimension remained in the range of 0.311–0.431), indicating that the studies were robust in this dimension. In the enterprise environmental behavior dimension, the effect sizes did not significantly vary within the range of 0.102 (i.e., the effect sizes of this dimension remained in the range of 0.507–0.609), indicating that the studies of this dimension were robust. In the enterprise social behavior dimension, the effect sizes did not significantly vary within the range of 0.165 (i.e., the effect sizes for this dimension remained in the range of 0.131–0.296), indicating that the studies were robust in this dimension. However, in the public participation dimension, the variation of the effect sizes reached 0.210 (i.e., the effect sizes of this dimension remained floating in the 0.308–0.518 interval). Apparently, the public participation dimension (with an effect size variation of 0.210) had the largest effect size variation range and was not robust compared to the three dimensions of enterprise economic behavior (with an effect size variation of 0.120), enterprise environmental behavior (with an effect size variation of 0.102), and enterprise social behavior (with an effect size variation of 0.165). After excluding the work of Liu et al. (2021) [34], the effect size of the public participation dimension was found to be 0.298. After excluding the work of Al-Kumaim et al. (2021) [59], the effect size of the public participation dimension was found to be 0.311. However, after excluding the work of Chen et al. (2020) [27], the effect size of the public participation dimension was found to be 0.476; for this reason, the work of Chen et al. (2020) [29] was excluded as an anomalous study.

## 4. Results and Discussion

### 4.1. Result of Meta-Analysis and Discussion

The results of the meta-analysis of the four variables corresponding to the mechanism of the role of government green development behavior are shown in Table 5. The results showed that government green development behavior was significantly and positively correlated with each dimension of enterprise behavior and the dimension of public behavior. The results of the study support hypotheses H1–H4. In general, the impact of government green development behavior was found to be positive. From the perspective of the main body, the effect of the public being influenced by government green development behavior was more obvious. From the perspective of sub-dimensions, enterprise environmental behavior presented the greatest relationship strength.

How do enterprises perform in the role of government green development actions? Enterprise economic behavior (0.411), enterprise environmental behavior (0.536), and enterprise social behavior (0.201) were shown to be significantly and positively correlated. According to neo-institutional theory, enterprises are subject to both normative and coercive institutional pressures from the government. For normative institutional pressure, previous scholars have mainly focused on the enterprise perspective and emphasized the positive effects of government green development behaviors (e.g., government environmental regulation) on enterprise economic behavior [63], enterprise environmental behavior [64], and enterprise social behavior [65]. This shows that the above studies support the results of this study. In terms of coercive institutional pressures, the results of this study are consistent with those of [18,66].

On the whole, enterprises are likely to respond with this response mechanism when they are constrained by government green development behavior. The root cause of this situation is that enterprises cannot afford to adopt behaviors such as resistance and decoupling in the face of institutional pressure from the government. On the economic level, according to the 2020 Annual Report on Ecological and Environmental Statistics published by the Chinese Ministry of Ecology and Environment [67], the fines for administrative penalties for environmental pollution behavior exceeded 8.24 billion yuan in 2020. Individually, there were gaps in the degree of effect of each dimension of enterprise behavior. Government green development behavior was found to have the greatest degree of effect on enterprise environmental behavior (0.536). Conversely, enterprise social behavior exhibited the smallest degree of effect (0.201). There are two reasons for this phenomenon. First, more people within business organizations are aware of the importance of environmental performance. Business operations revolve around performance as a core component, and business performance is usually measured with a combination of environmental, social, and economic aspects. Enterprise environmental behavior can be translated into environmental performance and thus into benefits for an enterprise. In addition, enterprise environmental performance can significantly and positively affect financial performance across time [68]. Second, the heterogeneity of enterprises leads to the adoption of social behaviors. In terms of enterprise social responsibility, the heterogeneity of enterprises lies in their size, industry, and nature. For example, listed enterprises regularly publish ESG (Environmental, Social, Governance) reports driven by external pressure and internal willingness. Moreover, a study by Lu et al. [69] showed that stakeholders have little influence on enterprise environmental behavior.

The result that government green development behavior significantly and positively influences public participation is consistent with the findings of scholars such as Chen et al. [29] and Chu et al. [70]. Environmental governance cannot be achieved without the efforts of stakeholders. Therefore, appropriate green development behaviors by the government can drive the public to participate in the environmental governance process and add to environmental governance.

### 4.2. Subgroup Result and Discussion

To investigate the difference in the extent of the role of green development behaviors of local and central governments, the authors of this study divided those effect values into two groups according to different subjects for subgroup analysis. Central government green development behavior was found to correspond to 12 effect values, and the local government green development behavior was found to correspond to 4 effect values. The results of the subgroup analysis shown in Table 6 indicate that both local and central government green development behaviors are significant. Further, the effect size of the green development behavior of local government (0.441) was found to be larger than that of central government (0.510). This result supports hypothesis H5. Possible reasons for this phenomenon are differences in the interpretation of policies and behavioral paths (e.g., incentives and regulatory intensity) between central and local governments. Central governments regulate and introduce policies from a macro perspective, while local governments develop specific measures based on policies that are more in line with local realities to achieve local green development.

### 4.3. Moderating Effects Result and Discussion

The results of the effect analysis of the moderating variables are shown in Table 7. As seen in the table, the effect sizes of China (0.524, *p* < 0.001), Pakistan (0.645, *p* < 0.001), and Turkey (0.244, *p* < 0.001) had a significant positive relationship. The results of this study suggest that there is a moderating effect of the region in government–enterprise and government–public interactions, thus supporting hypothesis H5. There are differences in the economic, political system, technology, energy, and green development levels in different regions, and this differentiation acts as a moderating effect between government–enterprise and government–public interactions.

### 4.4. Management Implication

How a government balances economic growth and environmental protection is a key concern for all sectors of society. By revealing the mechanism of government green development behavior, the authors of this study analyzed the government–enterprise and government–public relationships from the perspective of green development. Additionally, this study provides new ideas for improving the level of governmental green development. The authors of this study provide the following management suggestions for central and local governments to practice green development behavior.

For central governments, the authors of this study make the following specific recommendations. First, central governments play a macro-regulatory role by introducing more targeted laws and regulations and setting more appropriate environmental regulations. For example, heavily polluting industries such as building materials and chemicals are key targets of environmental management. These industries often bear heavier compliance costs than other industries in the process of green transformation. Therefore, central governments should appropriately increase subsidies and tax incentives when formulating relevant policies, which will reduce the compliance costs of enterprises and promote the formation of a parallel situation of economic development and environmental protection in society. Second, central governments also need to continuously improve the central government–local government and central government–enterprise regulatory systems. At the local government level, central governments should actively optimize the hierarchical structure to clarify the boundaries of the responsibilities of each level of government. In addition, central governments should send central environmental inspection teams from time to time to monitor the environmental management work of local governments. At the enterprise level, enterprises react differently when faced with pressure and constraints, such as obedience, resistance, and decoupling. Central governments need to focus on identifying the true reactions of enterprises in the regulatory system to prevent them from adopting speculative behaviors that hinder the green development of society. Third, central governments’ active role in guiding environmental protection is an essential part of creating a comprehensive green development environment. This ensures that local governments implement green development mandates and that enterprises take the initiative to fulfill social and environmental responsibilities. Central governments should strive to promote the public’s ability to more actively participate in green development.

For local governments, the authors of this study make the following specific recommendations. First, local governments should actively cooperate with central governments and deeply understand environmental policies and regulations. On this basis, they should refine and adjust such policies and regulations according to the actual local conditions. In this process, rewards and punishments should be dynamically adjusted to motivate enterprises and the public to participate in green development actions to achieve local adaptation. For example, in regions with high levels of green development, local governments can focus on green technology innovation. Local governments can use the subsidy mechanism of environmental regulation to encourage local enterprises and the public to participate in the innovation process. In less developed regions, governments can increase the environmental regulation of enterprises and carry out green development-themed publicity activities to raise public awareness of the environment. Third, local governments can take advantage of their proximity in the regulatory process to strengthen the intensity and frequency of supervision.

## 5. Conclusions

Based on stakeholder theory and neo-institutional theory, the authors of this study constructed a theoretical integration model of governmental green development behavior that includes government, enterprises, and the public. Then, meta-analysis was used to explore 18 high-quality empirical studies from 2012 to 2021 to analyze the mechanism of governmental green development behavior. The authors of this study also explored and verified the moderating effect of the green development behavior of central and local governments.

The main findings of this study are as follows:

(1) Enterprise economic behavior, enterprise environmental behavior, enterprise social behavior, and public participation are all significantly and positively influenced by the green development behavior of governments.
(2) Local government green development behavior has a greater binding effect than that of central governments.
(3) Regional heterogeneity moderates the relationship between government green development behavior, enterprise behavior, and public behavior.

The theoretical contribution of this study the enrichment of the research field of the stakeholder and neo-institutional theories, as the authors of this study explored the mechanism of the role of governmental green development behavior considering multiple subjects based on stakeholder and neo-institutional theories. Additionally, the findings of this study can be applied to related studies on governmental green development behavior. In addition, the authors of this study have provided a new understanding of stakeholder and neo-institutional theories by systematically integrating research on government green development behavior using meta-analysis.

This study also had some limitations. First, the authors of this study used the social system as the boundary and only studied the government, the public, and enterprises as the three main subjects. Future research can further subdivide these three subjects. Second, due to the limitations of the sample, the authors of this study only established a moderating effect. Future research could consider further exploring other potential moderating variables, such as enterprise size, enterprise nature, enterprise competition, and public category.

## Figures and Tables

**Table 1:** Research gap.

Researcher	Central Government Green Development Behavior	Local Government Green Development Behavior	Enterprise Economic Behavior	Enterprise Environmental Behavior	Enterprise Social Behavior	Public Participation
Zailani et al. (2012) [30]	√			√		√
Agan et al. (2013) [31]	√		√	√	√	√
Guo et al. (2017) [32]	√			√		
Chen et al. (2020) [29]	√					√
Huang et al. (2021) [33]		√		√		
Liu et al. (2021) [34]		√				√
This research	√	√	√	√	√	√

**Table 2:** Target literature code table.

	Author Year	Outcome	Sample Size	Fisher’s Z	Standard Error	Subject	Region
1	Wu (2017) [36]	EEnvB ^1^	227	0.388	0.067	local	China
2	Jiménez-Parra et al. (2018) [52]	ESB ^2^	213	0.104	0.069	central	various
3	Guo et al. (2017) [32]	EEnvB	60	0.822	0.132	local	China
4	Zhang et al. (2020) [53]	EEnvB	298	0.873	0.058	central	China
5	Liu et al. (2021) [34]	PP ^3^	6309	0.519	0.013	local	China
6	Aboelmaged (2017) [54]	EEconB ^4^, EEnvB	238	0.182, 0.090	0.065, 0.065	various	Egypt
7	Chen et al. (2020) [29]	PP	544	0.121	0.043	central	China
8	Ramírez et al. (2018) [55]	EEconB	407	0.587	0.050	central	Spain
9	Khan et al. (2021) [56]	EEconB	214	0.267	0.069	central	USA
10	Zailani et al. (2012) [30]	EEnvB	132	0.393	0.088	central	Malaysia
11	Agan et al. (2013) [31]	ESB, EEconB, EEnvB	500	0.102, 0.329, 0.300	0.045, 0.045, 0.045	central	Turkey
12	Huang et al. (2021) [33]	EEnvB	270	0.331	0.061	local	China
13	Li et al. (2020) [11]	EEnvB	615	0.511	0.040	central	China
14	Pham et al. (2021) [57]	ESB	137	0.210	0.086	central	Vietnam
15	Shahzad et al. (2020) [58]	ESB	318	0.353	0.056	central	Pakistan
16	Al-Kumaim et al. (2021) [59]	PP	300	0.499	0.058	central	Malaysia
17	Xue et al. (2021) [60]	ESB	360	0.081	0.053	central	China
18	Wen et al. (2019) [61]	EEnvB	288	0.968	0.059	central	Pakistan

^1^ EEnvB = Enterprise Environment Behavior; ^2^ ESB = Enterprise Social Behavior; ^3^ PP = Public Participation; ^4^ EEconB = Enterprise Economic Behavior.

**Table 3:** Bias test.

Outcome	Rosenthal’s Fail-Safe N			Begg and Mazumdar Rank Correlation *p*-Value	Egger’s Regression (2-Tailed)		
	z-Value	*p*-Value	α		*p*-Value	Low Limit	Upper Limit
Enterprise economic behavior	12.910	<0.001	0.050	0.497	0.484	−48.758	32.631
Enterprise environmental behavior	24.648	<0.001	0.050	0.835	0.782	−12.253	15.654
Enterprise social behavior	6.271	<0.001	0.050	0.327	0.687	−13.169	17.437
Public participation	30.379	<0.001	0.050	0.602	0.535	−90.684	78.732

**Table 4:** Total sample heterogeneity test.

Model	k	Combined Effect Size	95% Confidence Interval		Q-Value	df	*p*-Value	I^2^	τ^2^
			Low Limit	Upper Limit					
fixed	21	0.438	0.420	0.455	457.122	20	<0.001	95.625	0.047
random	21	0.379	0.282	0.476					

**Table 5:** Result of meta-analysis.

Category	Outcome	k	Combined Effect Size	95% CI		*p*-Value	Total Effect Size
				Low Limit	Upper Limit		
Enterprise behavior	enterprise economic behavior	3	0.411	0.353	0.470	<0.001	
	enterprise environmental behavior	8	0.536	0.496	0.576	<0.001	0.433
	enterprise social behavior	3	0.201	0.137	0.265	<0.001	
Public behavior	public participation	2	0.518	0.494	0.542	<0.001	0.518

**Table 6:** Subgroup analysis results.

Group	k	Effect Size	95% CI		Q-Value	df	*p*-Value	I^2^	τ^2^
			Low Limit	Upper Limit					
Central	12	0.441	0.411	0.472	236.421	11	<0.001	95.347	0.060
Local	4	0.510	0.486	0.534	17.970	3	<0.001	83.306	0.01

**Table 7:** Moderating effect results.

Region	k	Effect Size	95% CI		Q-Value	df	*p*-Value	I^2^	τ^2^	Qw	Qb
			Low Limit	Upper Limit							
China	6	0.524	0.502	0.546	55.387	5	<0.001	90.973	0.020	128.198	138.408
Malaysia	2	0.467	0.372	0.562	1.001	1	>0.050	0.130	0	*** ^1^	*** ^1^
Pakistan	2	0.645	0.565	0.725	56.672	1	<0.001	98.235	0.186		
Spain	1	0.587	0.490	0.685	0	0	>0.050	0	0		
Turkey	3	0.244	0.193	0.295	15.137	2	<0.001	86.788	0.013		
USA	1	0.267	0.132	0.402	0	0	>0.050	0	0		
Vietnam	1	0.210	0.041	0.379	0	0	>0.050	0	0		

^1^ ***, *p*-Value < 0.001.

## Data Availability

Not applicable.

## References

[1] Shen L., Wu Y., Lou Y., Zeng D., Shuai C., Song X. (2018). What drives the carbon emission in the Chinese cities?—A case of pilot low carbon city of Beijing. J. Clean. Prod..

[2] International Energy Agency (2022). Global Energy Review: CO_2_ Emissions in 2021. https://www.iea.org/data-and-statistics/data-product/global-energy-review-co2-emissions-in-2021.

[3] BP Global (2021). Statistical Review of World Energy. https://www.bp.com/en/global/corporate/energy-economics/statistical-review-of-world-energy.html.

[4] International Energy Agency (2022). Policies Database. https://www.iea.org/policies?type=Regulation.

[5] World Bank Group (2020). Wastewater a Resource That Can Pay Dividends for People, the Environment, and Economies, Says World Bank. https://www.worldbank.org/en/news/press-release/2020/03/19/wastewater-a-resource-that-can-pay-dividends-for-people-the-environment-and-economies-says-world-bank.

[6] Kaza S., Yao L., Perinaz B., Van Woerden F. (2018). What a waste 2.0: A global snapshot of solid waste management to 2050. Urban Development.

[7] UNEP, UNEP DTU Partnership Emissions Gap Report. https://www.unep.org/zh-hans/resources/emissions-gap-report-2021.

[8] Li X., Huang Y., Li J., Liu X., He J., Dai J. (2022). The mechanism of influencing green technology innovation behavior: Evidence from Chinese construction enterprises. Buildings.

[9] Li X., Dai J., Li J., He J., Liu X., Huang Y., Shen Q. (2022). Research on the impact of enterprise green development behavior: A meta-analytic approach. Behav. Sci..

[10] Li X., Du J., Long H. (2020). Understanding the green development behavior and performance of industrial enterprises (gdbp-ie): Scale development and validation. Int. J. Environ. Res. Public Health.

[11] Li X., Du J., Long H. (2020). Mechanism for green development behavior and performance of industrial enterprises (gdbp-ie) using partial least squares structural equation modeling (PLS-SEM). Int. J. Environ. Res. Public Health.

[12] Li X., Du J., Long H. (2019). Dynamic analysis of international green behavior from the perspective of the mapping knowledge domain. Environ. Sci. Pollut. Res..

[13] Ren S., Li X., Yuan B., Li D., Chen X. (2018). The effects of three types of environmental regulation on eco-efficiency: A cross-region analysis in China. J. Clean. Prod..

[14] Cheng B., Huang J., Li J., Chen S., Chen H. (2022). Improving contractors’ participation of resource utilization in construction and demolition waste through government incentives and punishments. Environ. Manag..

[15] Zhou Q., Zhong S., Shi T., Zhang X. (2021). Environmental regulation and haze pollution: Neighbor-companion or neighbor-beggar?. Energy Policy.

[16] Demirel P., Iatridis K., Kesidou E. (2018). The impact of regulatory complexity upon self-regulation: Evidence from the adoption and certification of environmental management systems. J. Environ. Manag..

[17] Ji H., Miao Z. (2020). Corporate social responsibility and collaborative innovation: The role of government support. J. Clean. Prod..

[18] Jiang Z., Wang Z., Lan X. (2021). How environmental regulations affect corporate innovation? The coupling mechanism of mandatory rules and voluntary management. Technol. Soc..

[19] Xie L., Li Z., Ye X., Jiang Y. (2021). Environmental regulation and energy investment structure: Empirical evidence from China’s power industry. Technol. Forecast. Soc. Chang..

[20] Freeman R.E. (2010). Strategic Management: A Stakeholder Approach.

[21] Arora P., De Y. (2020). Environmental sustainability practices and exports: The interplay of strategy and institutions in Latin America. J. World Bus..

[22] Tapaninaho R., Heikkinen A. (2022). Value creation in circular economy business for sustainability: A stakeholder relationship perspective. Bus. Strategy Environ..

[23] Yi L., Li T., Wang X., Ge G., Zhang T. (2022). Corporate social responsibility performance evaluation from the perspective of stakeholder heterogeneity based on fuzzy analytical hierarchy process integrated topsis. Corp. Soc. Responsib. Environ. Manag..

[24] Du L., Feng Y., Lu W., Kong L., Yang Z. (2020). Evolutionary game analysis of stakeholders’ decision-making behaviours in construction and demolition waste management. Environ. Impact Assess. Rev..

[25] Mia P., Rana T., Ferdous L.T. (2021). Government reform, regulatory change and carbon disclosure: Evidence from Australia. Sustainability.

[26] Sun R., Wang H. (2020). Game analysis for stimulating the driving forces of government data open from the perspective of new institutionalism theory. J. Inf. Resour. Manag..

[27] DiMaggio P.J., Powell W.W. (1983). The iron cage revisited: Institutional isomorphism and collective rationality in organizational fields. Am. Sociol. Rev..

[28] Pache A.C., Santos F. (2013). Inside the hybrid organization: Selective coupling as a response to competing institutional logics. Acad. Manag. J..

[29] Chen J., Huang J., Huang X., Sun S., Hao Y., Wu H. (2020). How does new environmental law affect public environmental protection activities in China? Evidence from structural equation model analysis on legal cognition. Sci. Total Environ..

[30] Hanim Mohamad Zailani S., Eltayeb T.K., Hsu C., Choon Tan K. (2012). The impact of external institutional drivers and internal strategy on environmental performance. Int. J. Oper. Prod. Manag..

[31] Agan Y., Acar M.F., Borodin A. (2013). Drivers of environmental processes and their impact on performance: A study of Turkish SMEs. J. Clean. Prod..

[32] Guo L., Qu Y., Tseng M.-L. (2017). The interaction effects of environmental regulation and technological innovation on regional green growth performance. J. Clean. Prod..

[33] Huang Y.C., Chen C.T. (2021). Institutional pressure, firm’s green resources and green product innovation: Evidence from Taiwan’s electrical and electronics sector. Eur. J. Innov. Manag..

[34] Liu H., Zhu G., Li Y. (2021). Research on the impact of environmental risk perception and public participation on evaluation of local government environmental regulation implementation behavior. Environ. Chall..

[35] Bohlmann C., Krumbholz L., Zacher H. (2018). The triple bottom line and organizational attractiveness ratings: The role of pro-environmental attitude. Corp. Soc. Responsib. Environ. Manag..

[36] Wu G. (2017). Environmental innovation approaches and business performance: Effects of environmental regulations and resource commitment. Innovation.

[37] Zhang Y., Zhao Z. (2022). Environmental regulations and corporate social responsibility: Evidence from China’s real-time air quality monitoring policy. Financ. Res. Lett..

[38] Wu L., Ma T., Bian Y., Li S., Yi Z. (2011). The local environmental state in China: A study of county-level cities in Suzhou. China Q..

[39] Li Z., Hou Y., Cao J., Ding Y., Yuan X. (2021). What drives green development in China: Public pressure or the willingness of local government?. Environ. Sci. Pollut. Res..

[40] Al-Swidi A., Saleh R.M. (2021). How green our future would be? An investigation of the determinants of green purchasing behavior of young citizens in a developing country. Environ. Dev. Sustain..

[41] Xu J., Byrne J. (2020). Explaining the evolution of China’s government–environmental NGO relations since the 1990s: A conceptual framework and case study. Asian Stud. Rev..

[42] Walther B.A., Yen N., Hu C. (2021). Strategies, actions, and policies by Taiwan’s ENGOs, media, and government to reduce plastic use and marine plastic pollution. Mar. Policy.

[43] Wu L., Ma T., Bian Y., Li S., Yi Z. (2020). Improvement of regional environmental quality: Government environmental governance and public participation. Sci. Total Environ..

[44] Wang Y., Zhao N., Lei X., Long R. (2021). Green finance innovation and regional green development. Sustainability.

[45] Zhou B., Wang Q., Zhang C. (2022). Central–local governance gaps: The evolving differentiation of climate policies in China. Sustain. Sci..

[46] Zou X., Lei C., Gao K., Hu C. (2019). Impact of environmental decentralization on regional green development. J. Environ. Dev..

[47] Michael Borenstein L.V.H., Higgins J.P.T., Rothstein H.R. (2009). Front matter. Introduction to Meta-Analysis.

[48] Palumbo S.A., Robishaw J.D., Krasnoff J., Hennekens C.H. (2021). Different biases in meta-analyses of case-control and cohort studies: An example from genomics and precision medicine. Ann. Epidemiol..

[49] Zhao G., Geng Y., Sun H., Tian X., Chen W., Wu D. (2022). Correction to: Mapping the knowledge of green consumption: A meta-analysis. Environ. Sci. Pollut. Res..

[50] Michael Borenstein L.V.H., Higgins J.P.T., Rothstein H.R. (2009). How a meta-analysis works. Introduction to Meta-Analysis.

[51] Fisher R.A. (1915). Frequency distribution of the values of the correlation coeffients in samples from an indefinitely large population. Biometrika.

[52] Jiménez-Parra B., Alonso-Martínez D., Godos-Díez J.L. (2018). The influence of corporate social responsibility on air pollution: Analysis of environmental regulation and eco-innovation effects. Corp. Soc. Responsib. Environ. Manag..

[53] Zhang W., Sun B., Xu F. (2020). Promoting green product development performance via leader green transformationality and employee green self-efficacy: The moderating role of environmental regulation. Int. J. Environ. Res. Public Health.

[54] Aboelmaged M. (2018). The drivers of sustainable manufacturing practices in Egyptian SMEs and their impact on competitive capabilities: A pls-sem model. J. Clean. Prod..

[55] Ramírez R.R., Palos-Sánchez P.R. (2018). Environmental firms’ better attitude towards nature in the context of corporate compliance. Sustainability.

[56] Khan S.A.R., Ponce P., Thomas G., Yu Z., Al-Ahmadi M.S., Tanveer M. (2021). Digital technologies, circular economy practices and environmental policies in the era of COVID-19. Sustainability.

[57] Pham H., Pham T., Dang C. (2021). Barriers to corporate social responsibility practices in construction and roles of education and government support. Eng. Constr. Arch. Manag..

[58] Shahzad M., Qu Y., Zafar A.U., Ding X., Rehman S.U. (2020). Translating stakeholders’ pressure into environmental practices—The mediating role of knowledge management. J. Clean. Prod..

[59] Al-Kumaim N.H., Shabbir M.S., Alfarisi S., Hassan S.H., Alhazmi A.K., Hishan S.S., Al-Shami S., Gazem N.A., Mohammed F., Abu Al-Rejal H.M. (2021). Fostering a clean and sustainable environment through green product purchasing behavior: Insights from Malaysian consumers’ perspective. Sustainability.

[60] Xue J., He Y., Liu M., Tang Y., Xu H. (2021). Incentives for corporate environmental information disclosure in China: Public media pressure, local government supervision and interactive effects. Sustainability.

[61] Jun W., Ali W., Bhutto M.Y., Hussain H., Khan N.A. (2019). Examining the determinants of green innovation adoption in SMEs: A pls-sem approach. Eur. J. Innov. Manag..

[62] Michael Borenstein L.V.H., Higgins J.P.T., Rothstein H.R. (2009). Identifying and quantifying heterogeneity. Introduction to Meta-Analysis.

[63] Sun T., Feng Q. (2021). Evolutionary game of environmental investment under national environmental regulation in China. Environ. Sci. Pollut. Res..

[64] Wang Y., Hu J., Hu Y., Wang Y. (2022). Which is more effective: The carrot or the stick? Environmental policy, green innovation and enterprise energy efficiency—A quasi-natural experiment from China. Front. Environ. Sci..

[65] Liu Q., Li L. (2019). Spatial heterogeneity of government regulation, spatial distance and enterprise carbon information disclosure: An analysis based on the heavy pollution industry in China. Int. J. Environ. Res. Public Health.

[66] Tang Y., Zhu J., Ma W., Zhao M. (2022). A study on the impact of institutional pressure on carbon information disclosure: The mediating effect of enterprise peer influence. Int. J. Environ. Res. Public Health.

[67] Ministry of Ecology and Environment of the People’s Republic of China (2022). Eco-Environmental Statistics Annual Report. https://www.mee.gov.cn/hjzl/sthjzk/zghjzkgb/202205/P020220608338202870777.pdf.

[68] Horváthová E. (2012). The impact of environmental performance on firm performance: Short-term costs and long-term benefits?. Ecol. Econ..

[69] Lu Y., Abeysekera I. (2014). Stakeholders’ power, corporate characteristics, and social and environmental disclosure: Evidence from China. J. Clean. Prod..

[70] Chu Z., Bian C., Yang J. (2022). How can public participation improve environmental governance in China? A policy simulation approach with multi-player evolutionary game. Environ. Impact Assess. Rev..

